# A Framework for the Automatic Integration and Diagnosis of Building Energy Consumption Data

**DOI:** 10.3390/s21041395

**Published:** 2021-02-17

**Authors:** Shuang Yuan, Zhen-Zhong Hu, Jia-Rui Lin, Yun-Yi Zhang

**Affiliations:** 1 Department of Civil Engineering, Tsinghua University, Beijing 100084, China; yuans17@mails.tsinghua.edu.cn (S.Y.); huzhenzhong@tsinghua.edu.cn (Z.-Z.H.); jiarui_lin@foxmail.com (J.-R.L.); 2 Shenzhen International Graduate School, Tsinghua University, Shenzhen 518055, China

**Keywords:** building energy consumption, data integration, energy usage diagnosis, artificial neural network

## Abstract

Buildings account for a majority of the primary energy consumption of the human society, therefore, analyses of building energy consumption monitoring data are of significance to the discovery of anomalous energy usage patterns, saving of building utility expenditures, and contribution to the greater environmental protection effort. This paper presents a unified framework for the automatic extraction and integration of building energy consumption data from heterogeneous building management systems, along with building static data from building information models to serve analysis applications. This paper also proposes a diagnosis framework based on density-based clustering and artificial neural network regression using the integrated data to identify anomalous energy usages. The framework and the methods have been implemented and validated from data collected from a multitude of large-scale public buildings across China.

## 1. Introduction

Buildings consume 40% of global primary energy and contribute to in excess of 30% of total CO_2_ emissions [1], yet building energy usage efficiency is currently distant from the optimum, as it is estimated that as much as 16% of total energy consumption during building operation could be conserved through proper management [2]. Costa et al. [1] put the potential of systematic building management in reducing energy consumption between 5% and 30%. The pursuit of improving building energy usage efficiency has motivated the advent of installing sensors and other smart metering devices for the collection of building energy consumption monitoring data, the evaluation and analyses of which have proved crucial in detecting anomalous energy usage patterns and ameliorating energy usage strategies. However, despite the proliferation of sensing devices making monitoring building energy consumption behavior easier, heterogeneity across different BEMSes (Building Energy Management System) has been a consistent obstacle to the acquisition of data with satisfactory quantity and quality. This lack of systematic data extraction and integration methods from heterogeneous sources also left research on the integrated analyses of static building properties and dynamic monitoring data vacant.

Massive and quality data are a prerequisite for effective and efficient building energy consumption management [3]. Obviously, two categories of data are relevant in managing building energy usage, with one being dynamic energy consumption monitoring data, while the other being building static data. The primary sources of dynamic energy consumption data, which are time-series readings over a fixed time interval, are the sensors and smart meters physically installed within buildings, usually managed by BEMSes [2]. However, no unified data standard exists for BEMSes, and data schemes of BEMSes from different manufacturers are usually distinct and incompatible. It currently remains a conundrum to integrate energy consumption monitoring data from heterogeneous data sources [4,5]. Building static data, on the other hand, are attributes and parameters of buildings that are time-invariant, and Building Information Modelling (BIM) is a universal technology for its digitization, which provides a faithful digital description of the building, geometric and semantic data included. The difficulty in the integration of data from heterogeneous sources confined analyses of building energy consumption data to single buildings, and consequently rendered static data futile because of its invariability. Numerous analyses and diagnoses of energy usage have been performed on the scale of single buildings, and static data were not involved [6,7]. Logically, the static properties of buildings, such as building area, exterior design, insulation, etc. are relevant to energy usage decisions of buildings. Therefore, integration of energy consumption data over a multitude of buildings introduce static building properties as new independent variables, and reveal the potential to unveiling new patterns in energy usage not previously perceived. Currently, the interaction between dynamic and static data in building energy consumption analyses is limited, and the relationship between the building parameters and energy performance has not been fully explored [8].

Academia has devoted much effort to solving this problem recently, as some researchers have proposed the utilization of semantic web technology to enhance the interaction between BIM models and physical data [9,10,11]. These methods, however, use data from existing systems, while it is more desirable to devise a framework for the integration of isolated data islands, namely between BIM models and energy consumption monitoring data, and among heterogeneous monitoring data. Researches in building energy consumption diagnosis and anomaly detection, on the other hand, have been abundant, yet these researches fail to incorporate static building information, and the relevant patterns thereof.

In this research, a unified framework for the extraction and integration of BIM models and building energy consumption monitoring data has been proposed. A hierarchical data model for building energy consumption monitoring data has been established in the framework, and an integration method for raw monitoring data has been proposed by deducing conversion logic from static topological information, allowing for the standardization, and thus integration, among otherwise isolated data. On this basis, energy usage anomaly diagnosis methods have been devised using integrated static and dynamic data, which employs density-based clustering and artificial neural network regression.

The remaining part of this paper is organized as follows. Section 2 provides a review of related researches in the literature. Section 3 introduces the framework for the automatic extraction and integration of static BIM models, and dynamic energy consumption monitoring data. Section 4 describes the proposed methods to diagnose energy usage anomalies with the integrated data. Section 5 contains a case study on a manifold of large-scale buildings across China to validate the proposed framework and methods, as well as discussions on the results. Finally, Section 6 provides a brief summary, the conclusions, and projections of future works.

## 2. Literature Review

This paper presents the design of a unified framework for the extraction and integration of static building information, and dynamic building energy consumption monitoring data, as well as statistical learning methods based on the framework to diagnose energy usage anomalies. This section provides a review of researches related to the topic in literature.

### 2.1. Extraction and Integration of Energy Consumption Monitoring Data and Static Building Data

The primary sources of dynamic building energy consumption monitoring data are sensors and smart meters installed inside buildings. These devices usually collect data on a fixed time interval that are either direct utility consumption readings, such as power or water usage, or data that reflect the operational status of the building, including indoor environmental parameters such as room temperature, humidity, light intensity, etc. and operational parameters of Mechanical, Electrical, and Plumbing (MEP) equipment, e.g., the cooling water temperature of the air conditioning system, etc. These sensors and meters are usually managed by BEMSes, which monitor the energy usage status of buildings throughout the operation and maintenance phase in buildings’ lifecycles, and are sometimes embedded with simple strategies to adjust energy usage through actuators based on sensor and meter input [12]. However, no universal standards exist to guarantee the interoperability and standardized data exchanging among BEMSes, creating difficulty in integrating building energy consumption monitoring data from BEMSes of different manufactures [13]. An existing framework for the interoperability among smart grids is the National Institute for Standards and Technology (NIST) framework, which includes protocols and standards for information management for the interoperability of smart grid systems and devices [14], yet such standards have not been developed on building and sub-building levels [15,16]. Multiple data models and schemes have been proposed and implemented by BEMS manufacturers and researchers on building and sub-building levels, despite the lack of interoperation standards [16,17,18]. This research established a hierarchical model for the representation of building energy consumption monitoring data that covers multiple granularities based on summarizing existing models.

BIM models are carriers of static building information and digital representations of the physical and functional characteristics of buildings and attached facilities [19]. The universally accepted neutral standard for BIM is the Industry Foundation Classes (IFC) standard, allowing for the integration and representation of information from various disciplines and stages in the lifecycle of buildings [20]. Yalcinkaya and Singh [21] have identified the simulation and assessment of building energy performance as one of BIM’s possible applications. The integration of BIM with energy consumption monitoring data enables interoperability, visualization, automation, and integration with other systems [22]. Relevant researches are still in the nascent stage, but have seen an exponential growth in recent years [23]. Since building energy consumption monitoring data, and time-series sensor readings in general, are well-structured relational data conducive for Structured Query Language (SQL) queries, the most popular method for their integration with BIM is to either export or transform BIM data into relational databases through Application Programming Interfaces (APIs) [24,25,26,27] or using new schemas [28,29,30]. Some Researchers have developed specific query languages for BIM [31,32], or used the semantic web or ontology approach for the integration [8,33,34,35].

In summary, lack of interoperability among BEMSes is a prominent problem in building energy consumption management on the scale of building groups or manifolds, with comprehensive data models for building energy consumption data covering multiple granularities also scarce. The involution of BIM models in energy management were also mainly for visualization purposes, and the building contextual information has not been sufficiently utilized. This paper presents a framework for the extraction and integration of building energy consumption monitoring data and BIM data by establishing a hierarchical building energy consumption data model, and proposing the corresponding data construction method from raw readings of sensors by conversion logic deduction from BIM models. Thus, existing BEMSes could be incorporated into the framework by satisfying relevant requirements on sensors and BIM models.

### 2.2. Diagnostics and Anomaly Detection of Building Energy Consumption

Anomaly detection refers to the process of detecting abnormal events not conforming to expected patterns [36]. Anomalies are usually contextual, i.e., the same energy consumption value might be considered anomalous under certain circumstances but not under others. The primary mission of building energy diagnostics is to identify anomalous energy usage patterns. Anomaly detection and diagnostics in building energy consumption is a prominent topic and has been widely researched. Numerous researches have employed historical building energy consumption data to identify anomalies. Chou and Telaga [6] proposed a two-stage real-time anomaly detection system by comparing measures consumption against predictions. Janetzko et al. [37] proposed an unsupervised anomaly detection system that used time-weighted historical power consumption data to perform predictions. Wrinch et al. [38] identified anomalies by analyzing energy consumption data in a weekly moving sliding window. Hill et al. [39] proposed a modeling approach diagnosing anomalies using forward predictions, without considering contexts. Bellala et al. [40] proposed an unsupervised clustering-based algorithm to identify anomalies among low-dimensional embedded power consumption data. Zorita et al. [41] proposed a multivariate technique involving climatic data and building construction characteristics to model energy consumption. Fan et al. [42] proposed a framework for excavating novel patterns for building diagnostics using association rules.

Almost all techniques of statistical learning, both supervised and unsupervised, have been employed in building energy consumption diagnostics, as well as ensemble learning methods, employing multiple learning algorithms simultaneously [43,44]. Numerous researches have also been carried out on building energy consumption prediction, which could also be utilized for diagnostic purposes with minimal modifications [44]. In terms of granularity, the literature spans the range from components to whole buildings [7]. Yet, to the best of the authors’ knowledge, building energy consumption diagnostics across multiple buildings have never been performed in building diagnostics, and therefore the static information has not been properly addressed as part of the context of anomalies. On the basis of the data extraction and integration framework proposed, this research proposes building energy consumption diagnostics methods across multiple buildings, with static building information as an integral part of the context. Both unsupervised clustering and supervised Artificial Neural Network (ANN) regression methods have been proposed to address different scenarios.

## 3. Framework for the Automatic Extraction and Integration of Building Energy Consumption Monitoring Data and BIM Models

**Figure 1:**
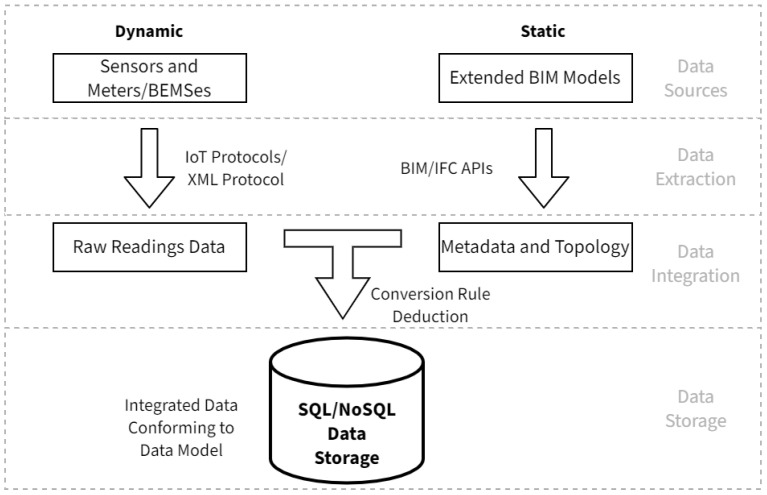
Architecture of the automatic extraction and integration framework for building energy consumption monitoring data with Building Information Modelling (BIM) models.

This paper proposes a unified framework for the automatic extraction and integration of building energy consumption monitoring data, as well as BIM models, as illustrated in Figure 1.

The model is constituted of four layers. The data sources layer contains sources for data in the framework, including sensors and smart meters for dynamic data, and extended BIM models for static data. The data extraction layer retrieves relevant data from the sources. For dynamic data, raw readings of the sensors and smart meters are extracted, which are stream monitoring data sampled by the sensors and meters at regular intervals. Existing BEMSes are also possible sources for dynamic data, through an Extensible Markup Language (XML) protocol proposed in this research. For static data, metadata for the sensors, as well as the building topology need to be extracted. conversion rules from raw readings to integrated data are deduced by topological Boolean operations in the Data integration layer, which performs the integration on the retrieved raw data following the deduced rules to convert raw sensing data into integrated data conforming to the data model. Finally, the data storage layer stores the integrated in both relational and NoSQL databases, and serves data to succeeding analyzing applications. Section 3.1, Section 3.2 and Section 3.3 explain the details of the framework design.

### 3.1. Hierarchical Building Energy Consumption Data Model and Data Storage

**Figure 2:**
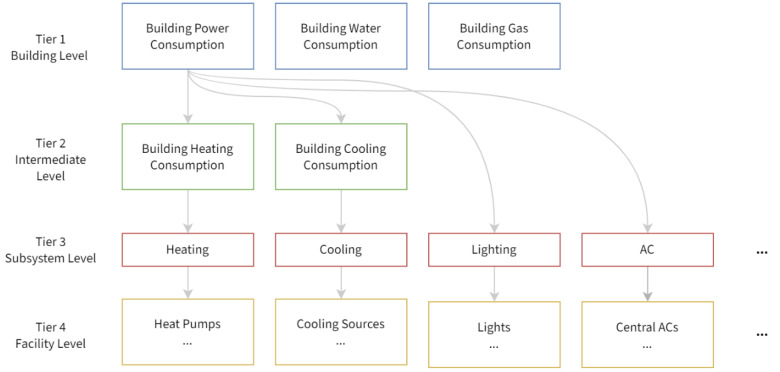
Illustration of the hierarchy of the building energy consumption data model.

The cornerstone of building energy consumption data analytics tasks, and all data analytics tasks in general, is to ascertain the data model, i.e., the inclusion and exclusion criteria of data, and their organization and description thereof. In this research, a generic hierarchical data model is proposed, whose hierarchy is illustrated in Figure 2, to achieve maximum compatibility across buildings, and cover the different granularities within.

The data model is organized as a four-tier tree hierarchy, each containing a number of utility consumption items corresponding to different granularities. The top tier contains three items corresponding the whole building granularity, i.e., the overall power, water, and gas consumption of the entire building. The top-middle tier contains the overall consumption of heating and cooling energy of the building, whose granularity is classified intermediate as the heating and cooling system implementation differ across buildings. For example, buildings in northern China usually have access to centralized heating in winter, where heating energy consumption is metered independently from power consumption, while buildings in southern china usually use air conditioning for heating purposes in winter, in which case heating energy consumption is essentially part of the overall electric power consumption. Organizing the heating and cooling energy of buildings into an intermediate level ensures compatibility with buildings of different heating and cooling system implementations. The bottom-middle tier corresponds to energy consumption records of building subsystems, including lighting, air conditioning, elevators, fans, etc. Lastly, the bottom tier contains records of the finest granularity—energy consumption of specific facilities. Each item contains 4 attributes, as shown in Table 1, that together uniquely identifies and describes a specific record.

**Figure 3:**
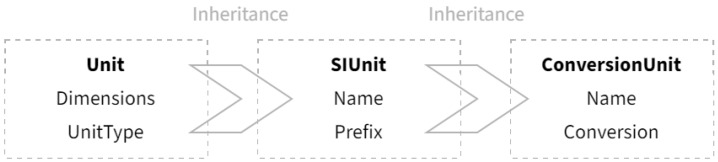
Illustration of the type system.

In the implementation of the framework, each energy item of each building would be assigned a code that uniquely identifies their combination, which is what the itemcode attribute stores. The time and value attributes are trivially designed to store the time and value of the energy consumption monitoring record. Finally, the unit attribute is of a simple type system specifically designed to represent the units of the records, as shown in Figure 3.

The type system is composed of three entities forming a single inheritance line. The base entity, unit, is an interface entity representing the type system containing a dimension array under the SI system (International System of Units, SI abbreviated from French Système International), and is inherited by the SIUnit entity, representing units that are decimal exponents under the SI system, such as joule, ton, cubic meter, etc. The SIUnit entity is in turn inherited by the ConversionUnit entity, representing units that are not convertible by decimal exponents under the SI system, such as kilowatt-hour, the most commonly used metric for power consumption. The inheritance chain ensures the Unit attribute, whose apparent type is the Unit base entity, could be bound to the correct underlying entity through the dynamic binding mechanism of Object-Oriented Programming (OOP). Table 2 demonstrates the connotations of the attributes in Figure 3, as well as an exemplification for the entity representing kilowatt-hour.

In this way, the proposed data model is complete and capable of representing energy consumption data across multiple buildings and granularities. The design of the data model, in general, is straightforward. As has been noted previously, multiple data models for the representation of building energy data have been proposed and implemented before, yet this data model has the advantage of the capacity for multi-building and multi-granularity representation, and therefore more suitable for data integration purposes. The design of the data model is in part based on the ontological building energy consumption data representation by Zhang et al. [45] Implementations of the framework could store the data in either relational databases or NoSQL databases depending on application scenarios. In general, relational databases are more intuitive and preferable in lightweight applications. NoSQL databases have scalability advantages and thus are more suitable for big data applications. There are also available methods for the storage of BIM data in both relational and NoSQL databases. This research implemented a NoSQL database for the integrated energy consumption data and BIM models, using the techniques devised by Lin et al. [46].

### 3.2. Extraction Methods of Energy Consumption Monitoring Data and BIM Models

The unified data model provided a criterion for data inclusion for further analysis, and a standardized way to organize and describe them. However, in the data sources side resides substantial heterogeneity, as numerous classification schemes and data models have been implemented by the existing BEMSes. In contrast, there exists the IFC standard as a universally accepted neutral standard for BIM models. To extract valid data for the generation of standardized data conforming to the data model, this research identifies and proposes compulsory static data requirements. Extraction techniques are then discussed, including an XML protocol devised for exporting data from existing BEMSes.

Standardized classification of sensors and smart meters is a prerequisite for the extraction of valid dynamic data, such that the same physical quantity is encoded identically across buildings. Table 3 lists the primary physical quantities measured by sensors recognized in this research, with each assigned a code. In this way, sensors and meters in each building could be parameterized uniformly. Note that Table 3 is not exhaustive.

On this basis, sensor and meter metadata in BIM models could be standardized. Besides the classification code of the physical quantity measured by the sensor, another attribute the entities for sensors and meters are obliged to possess is the facility or subspace whose energy consumption is being measured. Together with other necessary information, such as the name, identifier, and data transmission protocol, sensors could be embedded in BIM models through the extensibility mechanism of IFC. Such extended BIM models constitute the data requirements for buildings to be incorporated into the framework. With the proliferation of BIM technology applications, this requirement would be satiable by more and more buildings in the future. Large quantities of tools and APIs readily exist for extracting BIM and IFC data, which could be used to extract the sensor metadata and building topologies, to be used in the data integration layer.

**Figure 4:**
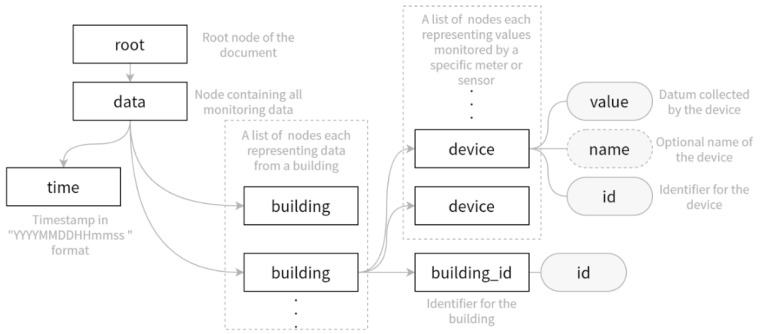
Graphical representation of the Extensible Markup Language (XML) protocol.

Data extraction for the dynamic part would be more straightforward, as sensor and meter readings could be collected through their respective data transmission protocol. Common IoT protocols include ZigBee, MQTT, Bluetooth, and Wi-Fi etc., which all have readily available data extraction tools. To utilize data in existing BEMSes, this research also proposed an XML interface for exporting BEMS data, as shown graphically in Figure 4.

The XML interface, as shown in Figure 4, has the data node within the root node, which contains the chunk of the data, including a timestamp indicating the time when the data are sampled, and a list of buildings whose data are further recorded in the device children nodes. The id property of the device node provides a handle to the meter entity in BIM models, thus a method to query the space or equipment to which the sensor or meter is bounded through the static data models. Thus, the interface carries all the necessary information to indicate the value and energy item of a raw monitoring record.

It has been one of the rising concerns in recent years that identifying information of citizens has been surreptitiously solicited and abused with the advent of data collection practices, thus violating privacy rights. In this research, the designs of the data model and the data collection procedures from the sensors included lists of physical quantities to be collected, among which only energy and resource consumption information of buildings and MEP systems are included. This information, though possibly could be used for building occupancy deduction, contains no indications for the identities of occupants.

### 3.3. Integration Method for Energy Consumption Monitoring Data and BIM Models

**Figure 5:**
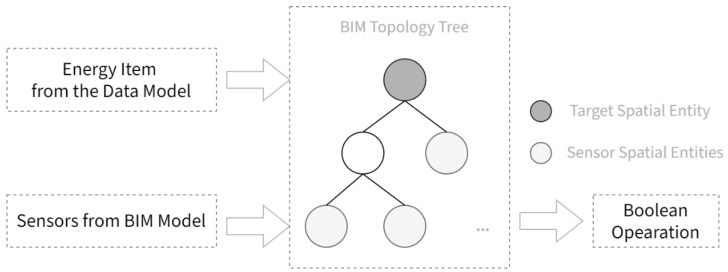
Raw data conversion formula deduction method from topology data.

The last remaining task in the framework is to convert and integrate raw readings extracted from the sensors and smart meters or BEMSes to data conforming to the universal data model described in Section 3.1. Since the sensors and meters might have different reading intervals, all the raw readings would first be aligned to sharp hours with a 1-h interval through linear interpolation. The key to the integration resides in the metadata requirements described in Section 3.2, as this research proposes a method to deduct conversion relationships between raw data and the data model from using the static data. The method is illustrated below in Figure 5.

The goal of the method is to deduce a formula for the generation of each energy item in the data model, e.g., the generation formula of the overall electric power consumption of a specific building. Therefore, the spatial entity corresponding to the item is first identified in the topology tree of the building, which was extracted from the BIM model. For example, the spatial entity corresponding to the overall power consumption of the building is the topological entity representing the entirety of the building. Then, spatial entities of all sensors in the building that monitor the relevant physical quantity are identified in the topological tree. In the case of electric power consumption, all meters and sensors monitoring positive active electric energy are involved. The final step is to generate a Boolean operation formula that takes sensor spatial entities and yields the target entity, using valid Boolean operations (i.e., union, intersection, and difference), which could be trivially converted to a formula of the readings of the sensors. Thus, formulae for the conversion are deducted, and hence the framework completes.

## 4. Diagnostics Methods with Integrated Dynamic and Static Data for Energy Usage Anomalies

An abundance of information is deposited in building energy consumption data, and extraction of such knowledge through appropriate analysis methods prove invaluable to optimizing building energy consumption management decisions. The identification of anomalies in building energy usage, or building diagnostics, is one of the most important and most researched applications building energy consumption data analytics. Since anomalies are the data points that do not conform to an expected pattern or behavior, anomaly detection tasks are essentially the learning of the underlying patterns of building energy consumption.

Unsupervised learning refers to the learning methods on data with unknown ground truth. Among unsupervised learning methods, clustering has been most frequently employed for anomaly detection tasks, with most researchers performing k-means clustering on building historical energy consumption data. K-means clustering requires as input the number of clusters to group the input data into, and groups data points into clusters of hyperspheres in the metric space. However, the number of clusters is hard to estimate beforehand, and the data points produced by the same energy usage pattern are not always hyper-spherical in the metric space. Therefore, an unsupervised learning approach using density-based clustering is proposed in this research, to eliminate explicit anomalies in the monitoring data.

Unlike unsupervised learning, supervised learning methods are trained on labeled data, and ANN is one of the most powerful and popular models for supervised learning. ANN is capable of regressing non-linear and high-order patterns among the input and output, is most often used to make predictions in building energy consumption data analytics. Authentic data are then compared against forward predictions for diagnostics, where data points whose deviation from the prediction exceeds a threshold are considered anomalous. Building static data, or building context, logically, are relevant to building energy consumption, yet have never been the subject of regressions against energy consumption data in the researches, since previous researches have all been performed on the level of single buildings or lower, where static data are invariant. On the basis of the framework described in Section 3, this paper proposes an ANN model regressing over input data from a multitude of buildings, with building static data treated as input parameters. This ANN model is used to identify implicit anomalies that are beyond the scope of clustering methods.

### 4.1. Density-Based Clustering Method for Explicit Anomaly Detection

**Figure 6:**
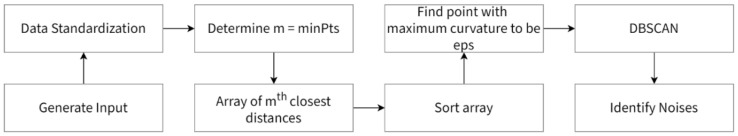
Flowchart of the proposed clustering algorithm.

Explicit anomalies are anomalies caused by errors in the facilities or sensing devices, are usually apparent to building managers. For example, negative reading of electric energy consumption would be classified as explicit. Clustering methods are more advantageous in identifying explicit anomalies than supervised learning methods as they are devoid of the problem of overfitting. This research proposes a density-based clustering algorithm to identify explicit anomalies in building energy consumption data, which includes the following 4 steps. The flowchart of the algorithm is as shown in Figure 6.

(1) Input Generation

Since the goal of this algorithm is to identify and eliminate explicit anomalies, it suffices to perform the analyses within buildings instead of across buildings. This method performs clustering over two-dimensional data, with cross-sectional historical data of the same energy item from the same time of day of the same building as one dimension, and temperature as the other, since it is the most significant meteorological factor affecting energy usage. Temperature records are available from local meteorological agencies.

(2) Data Standardization

Density-based clustering algorithms require as input a distance metric among data points, as well as a threshold for points to be considered neighbors. To diffuse the difference in the unit measurements of the two dimensions, data standardization is performed on the input, to convert both dimensions into standard normal distributions.

(3) Parameterization and Clustering

It suffices to use Euclidean distance as the distance measurement since it is conservative. Another parameter to designate is the minimum number of points for a neighborhood to be considered cluster, denoted as m or minPts, usually one more than the number of clusters. The final and most vital parameter is the distance threshold for two points to be considered neighbors, known as the epsilon. In this research, the epsilon is determined using the knee locator. To identify the knee locator, first the distance to the mth closest point for each point has to be found and stored in an array. Then, the array is sorted indexed in ascending order. Finally, the point with the maximum curvature is found, and the distance thereof is taken as the epsilon. The last step in this part is to perform Density-Based Spatial Clustering of Applications with Noise (DBSCAN) [47] clustering with the determined parameters. DBSCAN algorithm considers points closer than epsilon to be neighbors, and neighborhoods with at least m points to be a valid cluster. Points not belonging to any neighborhood would be considered explicit anomalies.

### 4.2. Artificial Neural Network Regression for Implicit Anomaly Detection

**Figure 7:**
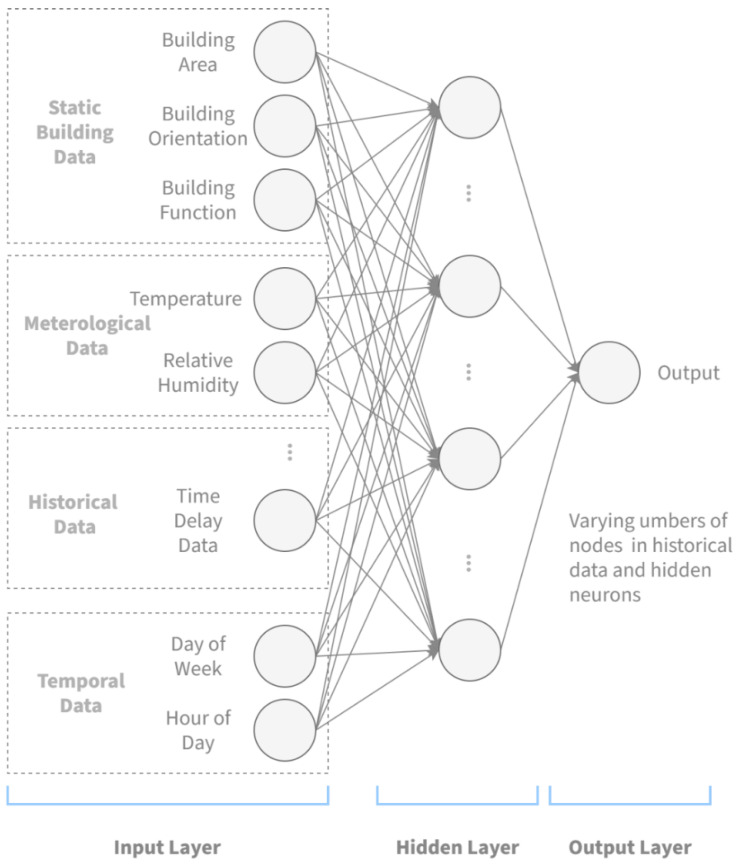
Structure of the artificial neural network.

Implicit anomalies refer to anomalies that are the result of changing energy usage patterns, which are usually not visually extruding. ANN is a powerful statistical learning method that potentially learns non-linear and high-order patterns in the input data, yet with known weaknesses in the tendency of overfitting. With explicit anomalies eliminated by the clustering algorithm described in Section 4.1, it follows that ANN could be utilized to diagnose implicit energy usage anomalies. The idea of anomaly detection with ANN is to train the network to make forward predictions, and use the predicted value as a benchmark for anomaly determination. To fully exploit the relevance between static building context and energy usage, this research proposes an ANN regression algorithm with building static properties as input. The network design is as shown in Figure 7.

The network model in this algorithm is a conventional multi-layered backpropagation neural network. It has three layers: an input layer, a hidden layer, and an output layer. The input layer includes a number of neurons representing a series of input parameters, divided into four groups. The first group represents the static building information, and comprises here of three parameters: building area, building orientation, and building function. The building area is valued as the total building area measured in square meters, and building orientation is denoted by the number of degrees it takes to rotate from due north to the direction the façade of the building is facing clockwise. The building function is represented using a dummy variable corresponding to an enumeration of building functionality. The second group contains meteorological data, and here includes the temperature in Celsius degrees and relative humidity in percentage points. Then, a number of historical energy consumption data immediately preceding the time to be estimated are organized in the third group, known as the historical data. Finally, the day of week, numbered 1–7, and hour of day, numbered 0–23, constitute the fourth group, the temporal data. The output layer has a single neuron for the dependent variable, i.e., the energy consumption to be estimated. The hidden layer has a number of neurons depending serving as relays between the input and output. Weights in the connections are generated at random from a gaussian distribution at initiation, and adjusted based on the error feedback once the training starts using Levenberg–Marquart backpropagation algorithm.

Since explicit anomalies have been eliminated previously by the clustering algorithm, overfitting should be expected at a minimum level. The logistic sigmoid function is used as the activation function in each neuron.

## 5. Case Studies and Discussions

### 5.1. Framework Implementation

**Figure 8:**
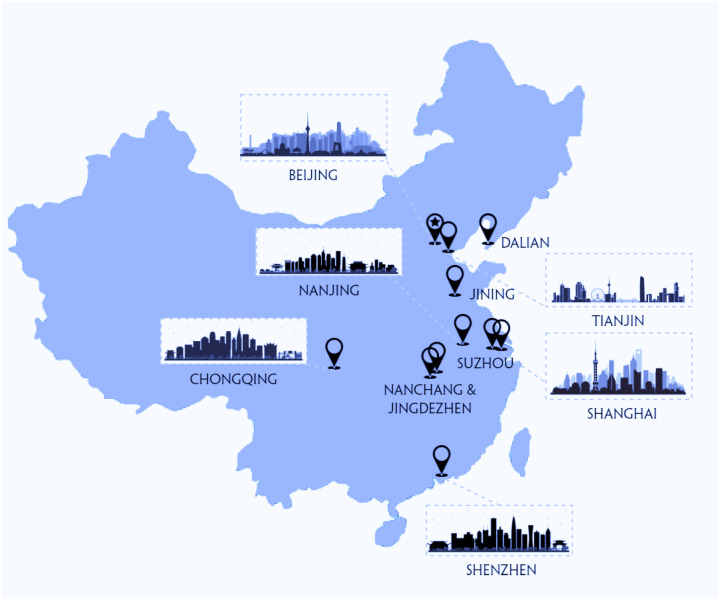
Geographic distribution of buildings included in the system.

To validate the extraction and integration methods, the universal data model, the framework has been implemented as part of an energy consumption management system construction effort. A total of 234 large public buildings from 11 cities across China have been incorporated into the framework after satisfying the metadata requirements, and a NoSQL database based on Hadoop has been deployed on the cloud end to store collected data. The sensors and meters in these buildings have been surveyed and tabulated following the data requirements described in Section 3, as well as built into corresponding BIM models through the extension mechanism of IFC. Figure 8 shows 11 cities pinned on the map where the 234 buildings are located.

**Figure 9:**
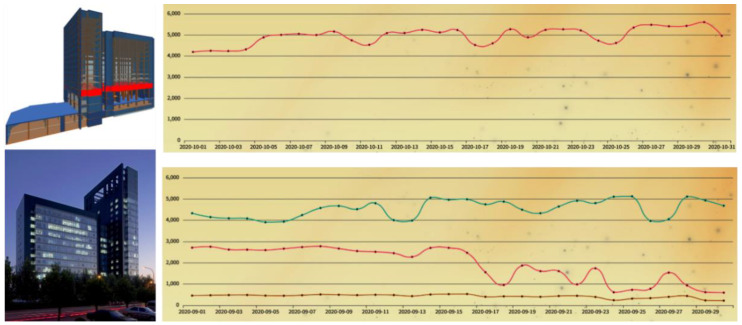
BIM model, photo, and monitoring data of Dalian University of Technology Innovation Park Building.

To date, the framework has been running for almost two years, covering a total building area of more than 12 million square meters. The sensors and smart meters collect information mostly every 5 min, and the raw data are integrated to be conforming to the data model with a time interval of 1 h. To date, more than 100 million integrated records have been accumulated in the storage, serving as valuable resources for not only diagnostics but numerous data analytics applications. Figure 9 shows the BIM model, outside photo, as well as monitoring data of one of the buildings, the Dalian University of Technology Innovation Park building. The monitoring data, plotted in the right half of the figure, are integrated records summarized daily in September 2020. The top right graph shows overall building power consumption, and the bottom right graph shows lighting (green), cooling (pink), and heating (brown) energy consumption in the building respectively. The unit of the vertical axis in both graphs is kilowatt-hour.

### 5.2. Testing of the Clustering Algorithm for Explicit Anomaly Detection

**Figure 10:**
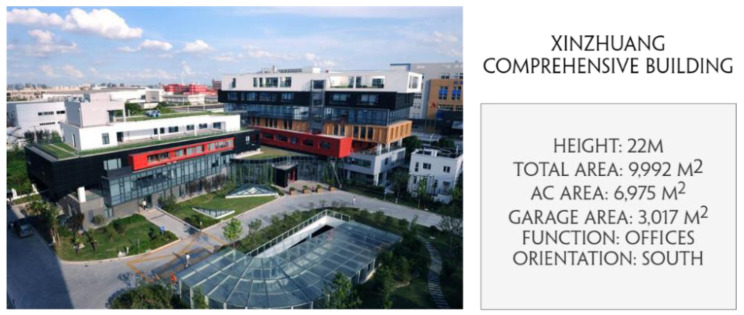
Information of Xinzhuang comprehensive building.

The density-based clustering algorithm has been validated using data from the Xinzhuang Comprehensive Building, a large-scale public building located in Xuhui district, Shanghai, China. The building measures 22 m in height, with seven floors aboveground, and one floor underground, totaling an area of 9992 square meters. Figure 10 is a photo of the building.

**Figure 11:**
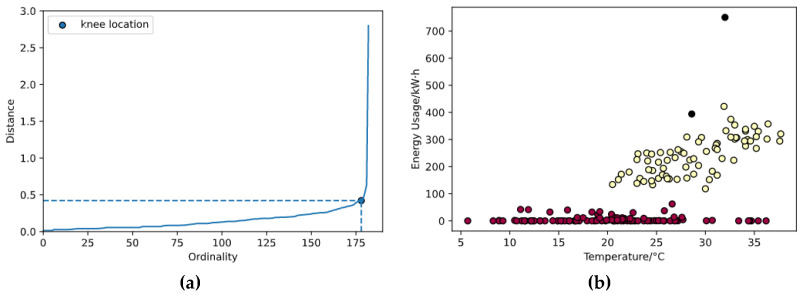
The knee locator and clustering result. (**a**) The knee locator; (**b**) The clustering result.

The input data for the algorithm are two-dimensional data comprised of outdoor dry-bulb temperature readings retrieved from local meteorological agencies, and the overall air conditioning subsystem power consumption of the building. Data at 15:00 each weekday from February to October 2020 are used. The data are then standardized so that both dimensions are converted to follow standard normal distributions. Euclidean distance is used as the distance metric for the standardized data points, while the minimum number of points for a neighborhood to be considered a cluster, m, is chosen to be 3, one more than the dimensionality. The epsilon is located using the knee locater, as described in Section 4, followed by DBSCAN clustering. Figure 11 shows the graph for knee locating, as well as the clustering result.

Figure 11a demonstrates the graph for knee locating, where the sorted array of 3rd closest distances is plotted against ordinals. The knee is defined as the point on the curve with the greatest curvature, or equivalently, the smallest radius of curvature. Figure 11b demonstrates the clustering result, where colored points represent clusters, and black points represent detected anomalies. The two detected anomalies are indeed visually explicit, and have been successfully identified. It could also be noted that the two clusters are of arbitrary shape instead of spherical, which implies k-means clustering would not work as ideally if applied.

### 5.3. Testing of the Artificial Neural Network Regression Algorithm for Implicit Anomaly Detection

**Figure 12:**
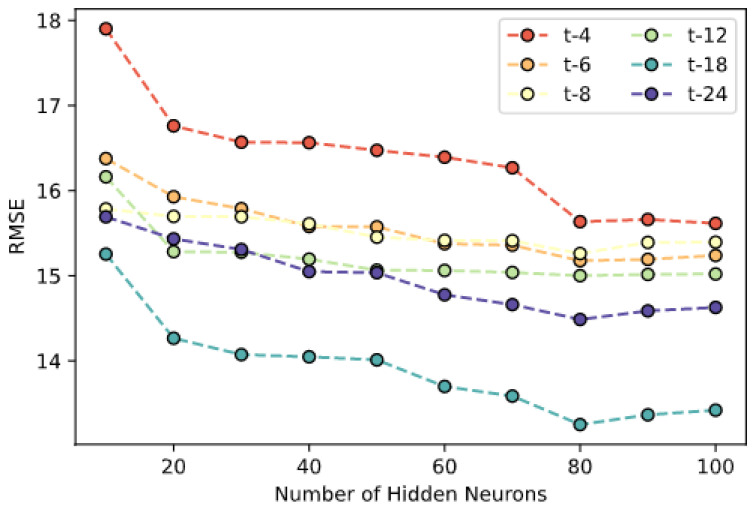
Influence of hidden neuron number and sliding window length on prediction results.

To validate the implicit anomaly detection method using artificial neural network regression with integrated static data, data from 10 large-scale public buildings in Shanghai are used to supply variability for static building information. There are shopping malls, office buildings, hotels, and hospitals among the 10 buildings, whose building function attributes are assigned the dummy values of 1–4 respectively. The overall power consumption data of the 10 buildings from November 2019 to October 2020 are used, with an interval of 1 h after integration (i.e., 24 records per building per day). Contemporary meteorological data are retrieved from local meteorological agencies, including dry-bulb temperature, and relative humidity. Temporal data that represents the day of week and hour of day are also generated. Six distinct Sliding window sizes between 4 and 24 have been used, and the number of hidden neurons varied between 10 and 100. The dataset was randomly divided into a training set and a testing set by a ratio of 9:1, and the training continues until improvement of loss is less than a preset tolerance of 0.0004. The prediction result is evaluated using Rooted Mean Squared Error (RMSE). Since the algorithm diagnoses anomalies through forwarding predictions, it follows that more accurate predictions on authentic data indicate better diagnostic performances. The influence of the number of hidden neurons and the length of the sliding window is shown in Figure 12.

Each line in Figure 12 represents the relationship between the RMSE of the prediction result on the testing set and the number of neurons in the hidden layer. It could be seen that the RMSE decreases steadily with the increase of neurons in the hidden layer before reaching 80, and remains mostly stagnant or slightly deteriorates thereafter, while a wider sliding window generally leads to better prediction results before reaching 18, especially under the circumstances where more hidden neurons are present. It could also be seen that the impact of sliding window width on the result is more significant and visible than the hidden neuron number. Therefore, in the following tests, 80 neurons are used in the hidden layer, and the width of the sliding window of historical power consumption data immediately preceding the time predicted is set to be 18.

**Figure 13:**
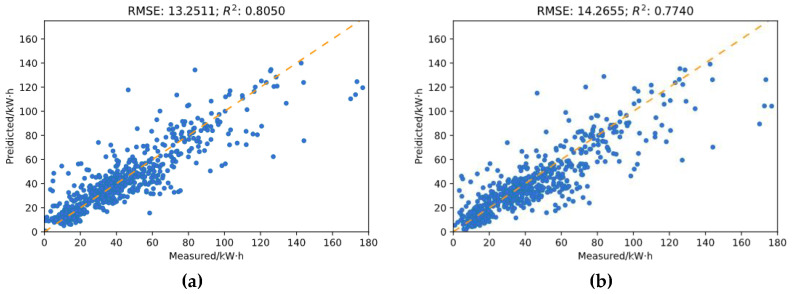
Comparison of the prediction results of the two models. (**a**) Prediction results from the network trained with static input; (**b**) Prediction results from the network trained without static input.

To test the validity of introducing static data in the input, a comparison experiment was performed. Two models were trained independently, with identical model parameter settings as described in the paragraph above. The same data are used as in the previous experiment, and the same train–test split was applied to the input of both models. The only distinction between the models is that one model has to build static data in the input, while the other does not. Figure 13 demonstrates the prediction results of the two models on the same testing set.

In Figure 13, the predicted power consumption values are plotted against the measured values, or the ground truth. Figure 13a shows the prediction result of the model with static data in the input, while Figure 13b shows the result of the other model. It could be noted that the prediction results of the two models are generally acceptable, as the points in the graphs are generally distributed along the diagonal line. The model with static data input outperforms the other with a moderate margin, as the RMSE of the prediction result is around 1 less than that of the other. Moreover, the coefficient of determination, or R^2^, of the regression on the left is greater than that on the right, indicating that additional energy consumption variations are explained by the static data.

### 5.4. Discussions

For the validation of the framework, the extraction and integration methods, and the diagnostics algorithms proposed in this research, an implementation of the system was presented in the case study, with more than 200 buildings accessed for data. The diagnostics algorithms have also been experimented, using data extracted and integrated into the framework. The achievement has supported the effectiveness and feasibility of the data extraction and integration methods. The experiment result of the explicit anomaly detection algorithm using density-based clustering also proved successful, as the visually explicit anomalies have been correctly identified and eliminated. Also, since the clusters in the result are of arbitrary shape, the performance of k-means clustering under the same circumstance could be put into question. Finally, the experiment result of the implicit anomaly detection method using ANN regression proved that the introduction of static data is of value, and improves the prediction result by a moderate margin. This, retrospectively, proves the value of the unified data extraction and integration framework, which made regression on the scale of building multitude possible.

This research, inevitably, is not devoid of vulnerabilities. The authors have identified the following problems that need further improvements.

(1) The feasibility of the extraction and integration methods depends on the quality of static metadata. Though BIM has been increasingly universal in the Architecture, Engineering, and Construction (AEC) industry, its application in building Operation and Maintenance (O&M) management is far from ubiquitous. Even under circumstances where the BIM model is present, the inclusion of the stipulated metadata in the model is not guaranteed, and costs extra time and labor to complement.
(2) Although the anomalies identified in the clustering experiment are visually explicit, there lacks a proper quantitative measurement for the clustering result. Intra-cluster similarity, in this case, is not well-defined, and an appropriate indicator remains to be worked out.
(3) The performance of the ANN could potentially be improved by normalizing input data, especially static building data, to accelerate convergence and prevent local optima stagnations. However, the normalization of input could be canceled out by linear transformations of weights in the process of training, thus theoretically input normalization does not affect training output. Due to the simplicity of the network employed in this research, the authors have also not found statistically significant improvements to the prediction accuracy by the introduction of input normalization. It is nonetheless noteworthy that in more complex networks, this could be overturned.
(4) The results of the ANN regression experiment, although demonstrated the effectiveness of the introduction of static data in the diagnostics, also manifested its magnitude of improvement is only moderate. The improvement in the prediction results cannot be guaranteed to justify the extra cost of implementing the framework. However, some more relevant static data were not available to the authors for the experiment, such as exterior transparency, insulation thickness or the coefficient of heat transfer of the walls, it is possible that further introduction of these variables would continuously enhance the prediction results. Moreover, from an absolute perspective, the error reported in this research is higher than in similar works than applied neural networks in time-series predictions. Despite the differences in data quality, network design and complexity, and input selection, this also signals the potential to further enhance prediction performances [48,49,50,51].

## 6. Conclusions and Future Works

Much research has been conducted on building energy consumption data, and sensing data in general, extraction and integration, and many practical BEMSes are implemented and installed in buildings. However, interoperability among heterogeneous BEMSes remains a problem, and a universal data exchange method for integrating energy consumption monitoring data from different buildings remains absent. Numerous studies have also been done on the diagnostics of building energy usage, with researches employing ANN particularly abundant. Yet, the problem of overfitting in supervised learning has not been overcome due to data noises, and building static data have never been considered for the lack of integrated dynamic and static data.

In this research, a framework for the automatic extraction and integration of building energy consumption monitoring data is proposed, containing a unified hierarchical data model for the description of building energy items covering different granularities, and corresponding data extraction techniques involving an XML protocol for exchanging data with BEMSes, and data integration techniques by conversion formulae deduction from topological data and metadata. On this basis, a density-based clustering algorithm along with the parameterization method was proposed to identify and eliminate explicit anomalies, to improve data quality for the succeeding regression. Then, an ANN regression method with building static data in the input is proposed to identify implicitly anomalous energy usage patterns. The methods and algorithms have been validated and tested in experiments, proving the value of the framework, and the effectiveness of the diagnostics methods.

For future research, the utilization of ensemble learning in the diagnostics combined with static input is a possible direction for further studying. Appropriate indicators for the clustering algorithm and further incorporation of more relevant static attributes are also worthy topics. Hardware implementations of the network would also be within consideration, should the performance of the methods improve further.

## Figures and Tables

**Table 1:** List of attributes of energy items in the data model.

Name	Type	Connotation
itemcode	string	Uniquely identifies the building and energy item
time	string	Time identifier formatted ‘YYYYMMDDHHmmss’
value	float	Value of the record
unit	Unit	Designates the unit of the record

**Table 2:** Connotation of attributes in the unit system with example.

Entity	Name	Type	Connotation	Exemplary Value
Unit	Dimensions	array [7]	Dimensional array under the SI system	(1, 2, −2, 0, 0, 0, 0)
	UnitType	enum	Physical quantity measured	UnitType::Energy
SIUnit	Name	enum	Name of the unit	UnitName::Joule
	Prefix	int	Exponent of 10 under the SI system	0
ConversionUnit	Name	enum	Name of the unit	UnitName::Kilowatthour
	Conversion	float	Conversion coefficient of the base unit	3600.

**Table 3:** List of primary physical quantities in the classification scheme.

Code	Physical Quantity Measured
U	voltage
UA, UB, UC	voltage of phase A, B, and C
I	electric current
IA, IB, IC	electric current of phase A, B, and C
WPP	positive active electric energy
APP, ATF	pressure, and discharge of compressed air
LIT, LRT	initial and return temperature of cooling water
PM10, PM2.5	concentration of PM10 and PM2.5 particles
TMP, HUM	temperature, and humidity
PH	pumping lift

## Data Availability

Data reported in this article are available upon request to the corresponding author.
